# Decoding Neural Activity in Sulcal and White Matter Areas of the Brain to Accurately Predict Individual Finger Movement and Tactile Stimuli of the Human Hand

**DOI:** 10.3389/fnins.2021.699631

**Published:** 2021-08-17

**Authors:** Chad Bouton, Nikunj Bhagat, Santosh Chandrasekaran, Jose Herrero, Noah Markowitz, Elizabeth Espinal, Joo-won Kim, Richard Ramdeo, Junqian Xu, Matthew F. Glasser, Stephan Bickel, Ashesh Mehta

**Affiliations:** ^1^Feinstein Institutes for Medical Research at Northwell Health, New York, NY, United States; ^2^Institute of Bioelectronic Medicine, Feinstein Institutes for Medical Research, New York, NY, United States; ^3^Hofstra-Northwell Medical School, New York, NY, United States; ^4^Department of Neurosurgery, Northwell Health, New York, NY, United States; ^5^Department of Radiology and Psychiatry, Baylor College of Medicine, Houston, TX, United States; ^6^Department of Radiology and Neuroscience, Washington University in St. Louis, St. Louis, MO, United States; ^7^Department of Neurology, Northwell Health, New York, NY, United States

**Keywords:** neuroprosthetics, stereoelectroencephalography, sensorimotor, tactile stimuli, neural decoding

## Abstract

Millions of people worldwide suffer motor or sensory impairment due to stroke, spinal cord injury, multiple sclerosis, traumatic brain injury, diabetes, and motor neuron diseases such as ALS (amyotrophic lateral sclerosis). A brain-computer interface (BCI), which links the brain directly to a computer, offers a new way to study the brain and potentially restore impairments in patients living with these debilitating conditions. One of the challenges currently facing BCI technology, however, is to minimize surgical risk while maintaining efficacy. Minimally invasive techniques, such as stereoelectroencephalography (SEEG) have become more widely used in clinical applications in epilepsy patients since they can lead to fewer complications. SEEG depth electrodes also give access to sulcal and white matter areas of the brain but have not been widely studied in brain-computer interfaces. Here we show the first demonstration of decoding sulcal and subcortical activity related to both movement and tactile sensation in the human hand. Furthermore, we have compared decoding performance in SEEG-based depth recordings versus those obtained with electrocorticography electrodes (ECoG) placed on gyri. Initial poor decoding performance and the observation that most neural modulation patterns varied in amplitude trial-to-trial and were transient (significantly shorter than the sustained finger movements studied), led to the development of a feature selection method based on a repeatability metric using temporal correlation. An algorithm based on temporal correlation was developed to isolate features that consistently repeated (required for accurate decoding) and possessed information content related to movement or touch-related stimuli. We subsequently used these features, along with deep learning methods, to automatically classify various motor and sensory events for individual fingers with high accuracy. Repeating features were found in sulcal, gyral, and white matter areas and were predominantly phasic or phasic-tonic across a wide frequency range for both HD (high density) ECoG and SEEG recordings. These findings motivated the use of long short-term memory (LSTM) recurrent neural networks (RNNs) which are well-suited to handling transient input features. Combining temporal correlation-based feature selection with LSTM yielded decoding accuracies of up to 92.04 ± 1.51% for hand movements, up to 91.69 ± 0.49% for individual finger movements, and up to 83.49 ± 0.72% for focal tactile stimuli to individual finger pads while using a relatively small number of SEEG electrodes. These findings may lead to a new class of minimally invasive brain-computer interface systems in the future, increasing its applicability to a wide variety of conditions.

## Introduction

There are currently 5 million people living with paralysis in the United States (Armour et al., 2016). The leading causes include stroke, spinal cord injury, multiple sclerosis, and amyotrophic lateral sclerosis (ALS). Brain-computer interface (BCI) technology was successfully demonstrated to form a neural bypass and restore volitional movement in paralysis during a first-in-human study that involved decoding movement-related neural signals recorded *via* microelectrodes implanted in motor cortex (Bouton et al., 2016). Furthermore, restoration of the sense of touch, important to regaining dexterous hand movement, has been demonstrated with BCI technology as well (Flesher et al., 2016). Microelectrodes and electrocorticography (ECoG) electrodes have been used for BCI applications, however, require a lengthy craniotomy to implant, thereby adding risk to the procedure. More recently, stereoelectroencephalographic (SEEG) electrodes have been used for mapping seizure origination in epileptic patients and is now widely accepted, offering a minimally invasive method for these procedures. SEEG electrodes are thin (< 1 mm) electrodes that are typically 25–30 cm in length and are inserted through a small hole (∼2.4 mm in diameter) made in the skull. This reduces the total area of the skull openings needed for SEEG electrodes significantly as compared to the large craniotomy area required for implanting micro- and ECoG electrodes. Adverse events associated with SEEG procedures occur at a significantly lower rate than with electrocorticography (ECoG) electrodes (Cardinale et al., 2013; Stricsek et al., 2018). SEEG electrodes therefore may be a good alternative to ECoG or microelectrode arrays in brain computer interface (BCI) systems.

Decoding performance in BCI systems is an important consideration and can be directly impacted by the location and type of electrode used. ECoG and microelectrode array, placed on the gyri, have been demonstrated in primates and humans for a variety of decoding applications including thought-controlled cursor movement and robotic arm control (Carmena et al., 2003; Hochberg et al., 2006). Decoding of individual finger movement has also been demonstrated in ECoG recordings (Kubánek et al., 2009). Very little work has been conducted to date, however, in assessing decoding performance when using SEEG electrodes for BCI applications. Basic two-dimensional cursor control has been demonstrated *via* SEEG electrodes (Vadera et al., 2013), in which the user wiggled their contralateral hand, or foot, to control the horizontal and vertical motion of a computer cursor, respectively. Also, a BCI P300 Speller (single degree-of-freedom) was controlled through ECoG and SEEG electrodes implanted in and near the hippocampus (Krusienski and Shih, 2011; Shih and Krusienski, 2012). Also, grasp force related events were recorded and classified using SEEG electrodes recording from sulcal areas in motor cortex and from sensory cortex (Murphy et al., 2016). Finally, SEEG provides access to subcortical areas including white matter. Recent studies showed white matter signals may contain a mixture of nearby and distant gray matter activity (Mercier et al., 2017). Also, high density EEG studies have also shown that sources involving sensorimotor processing can be located in white matter areas (Melnik et al., 2017). Furthermore, recent studies have also shown SEEG recording sites located in white matter can contribute to accurate decoding (Huang et al., 2019, 2021).

One compelling application of SEEG electrodes is in a so-called bidirectional neural bypass for restoration of movement and the sense of touch. Although activity related to arm kinematics has been found in the somatosensory area (Chowdhury et al., 2020), a minimally invasive approach is of particular interest where the bi-directional neural bypass application involves stimulating the somatosensory area. It has been shown that microstimulation of the primary sensory cortex can evoke tactile percepts that improve robotic arm control *via* a bidirectional BCI (Flesher et al., 2021). Specifically, precise stimulation of sulcal and subcortical areas for evoking highly focal percepts in the fingertips has also been demonstrated in humans recently using SEEG electrodes (Chandrasekaran et al., 2020). Furthermore, a bidirectional neural bypass needs to be effective in restoring a wide variety of movements including sustained movements to be useful for disabled users in activities of daily living. Previous work has shown that predominantly phasic (transient) neural modulation patterns were obtained during movements, but these studies were mostly centered on short or pulsed, but not sustained, movements (Shin et al., 2012; Chen et al., 2014; Flint et al., 2017). Also, recent work has further confirmed that predominantly phasic (transient) neural patterns in SEEG recordings occur during sustained grasping movements (Jiang et al., 2020). This suggests that using this type of activity as input features to a decoder for restoring sustained movement in an electronic neural bypass system may pose significant challenges. Another challenge stems from noise and amplitude drift/variation that occurs in neuronal activity over time which can degrade decoding performance (Zhang et al., 2018; Rule et al., 2020). This emphasizes the importance of using effective signal processing and feature selection methods that extract reproducible features for decoding. Furthermore, machine learning methods that can effectively use transient features while producing sustained outputs is important for restoring realistic and useful hand movements.

A number of strategies and different machine learning algorithms including linear classifiers, regression machines, support vector machines (SVMs), and deep neural networks have been used to decode neural signals recorded in the brain. In the handful of studies that have attempted to decode SEEG signals specifically, the results have been mixed. In one of these studies, three different hand gestures were decoded using SEEG signals with an accuracy of 78.70 ± 4.01% (Li G. et al., 2017). In another study, SEEG electrodes placed in middle temporal regions led to fast typing of up to 14 characters/minute (Li D. et al., 2017). Lastly, another group decoded SEEG recordings from the auditory cortex and produced intelligible waveforms with 45–75% accuracy levels depending on the algorithm used (Akbari et al., 2019).

Here we investigated the temporal characteristics of neural signals for both motor and sensory events when recorded using SEEG electrodes and decoding methodologies that incorporated temporal correlation-based feature selection and deep learning methods. Specifically, we were interested in decoding sustained finger movements and tactile stimuli and hypothesized long short-term memory (LSTM) based recurrent neural networks (RNNs), due to their memory cells and ability to handle temporal dependencies, would lead to sustained outputs and improved decoding accuracy despite receiving transient inputs (neural features). It was previously shown that a LSTM network can outperform a Kalman filter in a neural decoding application (Hosman et al., 2019). Furthermore, a LSTM network was specifically selected due to its improved performance, and ability to handle the vanishing gradient problem, over vanilla RNNs (Bengio et al., 1994; Shewalkar et al., 2019). Lastly, with a long-term goal of restoring dexterous hand movement, we investigated decoding performance for individual finger movement and touch-related events at the fingertips. Our findings show that neural patterns recorded with SEEG electrodes are indeed mostly phasic in nature, but that LSTM-based deep learning networks combined with repeatability-based feature selection can produce sustained outputs and high decoding accuracies.

## Materials and Methods

### Participants

**FIGURE 1:**
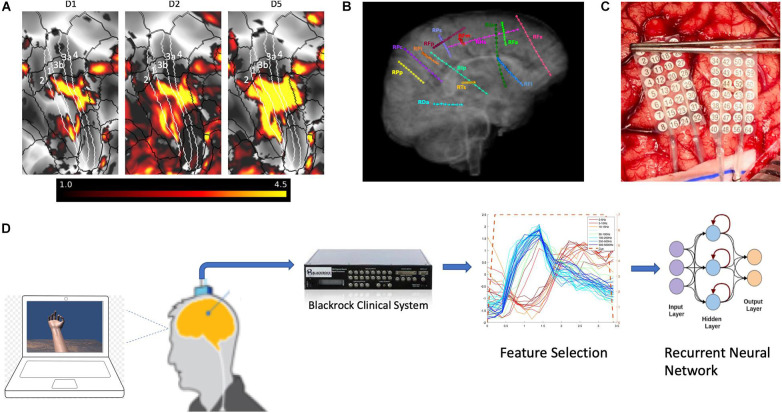
Functional magnetic resonance imaging and electrode placement. **(A)** Pre-surgical fMRI obtained while participant P1 pressed different buttons on a handheld device while watching videos showing desired movements. **(B)** Exemplary placement of SEEG electrodes (participant 3). **(C)** Photograph of HD ECoG electrode placement for recordings in participant P1_03. **(D)** Experimental setup where participants received visual cues of hand movements on a laptop computer with cues lasting 3 or 4s followed by a 3 or 4s period of rest; the clinical recording system (Natus Medical, Inc.) is not shown and was always connected for continuous data acquisition.

Three patients voluntarily took part in this study that were undergoing pre-operative seizure monitoring for surgical treatment of intractable epilepsy. Functional magnetic resonance imaging (fMRI) was performed in participants 1 and 3 (P1 and P3) and implantation of either HD-ECoG grids and/or SEEG electrode leads was performed in each participant, and signals were recorded during various motor and sensory tasks (see Figure 1). The decisions on whether to implant, the electrode targets selected, and the duration for implantation were based entirely on clinical grounds without reference to this investigation. Some patients required re-implantation which is indicated by an underscore after the participant code (e.g., _02, _03). Patients were informed that participation in this study would not alter their clinical treatment, and that they could withdraw from the study at any time without jeopardizing their clinical care. All procedures and experiments were approved by the Northwell Institutional Review Board and participants provided informed consent prior to enrollment into the study.

### Imaging

Participants were scanned on a 3T MRI scanner (Skyra, Siemens, Germany) with a 32-channel head coil. Human Connectome Project (HCP)-like structural and functional MRI were acquired: T1-weighted (T1w) 3D MPRAGE sequence, 0.8 mm isotropic resolution, TR/TE/TI = 2,400/2.07/1,000 ms, flip angle = 8 degree, in-plane under-sampling (GRAPPA) = 2, acquisition time 7 min; T2-weighted (T2w) 3D turbo spin echo (SPACE) sequence, 0.8 mm isotropic resolution, in-plane under-sampling (GRAPPA) = 2, TR/TE = 3,200/564 ms, acquisition time 6.75 min; task fMRI using the CMRR implementation of multiband gradient echo echo-planar imaging (EPI) sequence (Setsompop et al., 2012), 2.1 mm isotropic resolution, 70 slices with a multiband factor of 7 (Xu et al., 2013), FOV 228 mm × 228 mm, matrix size 108 × 108, phase partial Fourier 7/8, TR/TE = 1,000/35 ms, flip angle = 60 degree, phase encoding direction = anterior-posterior (A-P), echo spacing = 0.68 ms, 240 volumes in 4 min; and a pair of reversed polarity (A-P/P-A) spin echo EPI field mapping acquisitions with matched echo train length and echo spacing to the fMRI acquisition. The task was button-pressing on the PST button response unit (Psychology Software Tools, Sharpsburg, PA, United States) using a single finger (wrist restrained with strap on the button response unit and neighboring fingers taped down with medical tape), repeating 6 times of 20-second off (resting with cue of a blank dark screen) and 20-second on (tapping with continuous video cue of the same finger motion presented from a projector screen). Participant P1 performed three repetitions of the task for each of thumb, index, and little fingers (phase-encoding direction A- > P) while participant P3 performed two repetitions for each of thumb, index, and middle fingers (phase-encoding directions A- > P and P- > A). The MRI preprocessing began with the HCP minimal preprocessing pipelines version 3.27 (Glasser et al., 2013) including, motion correction, distortion correction, cortical surface reconstruction and subcortical segmentation, generation of T1w/T2w-based myelin content and cortical thickness maps, transformation of the fMRI data to MNI and CIFTI grayordinate standard spaces using folding-based registration with MSMSulc, and 2 mm full-width half maximum (FWHM) surface and subcortical parcel constrained smoothing for regularization. The fMRI data were cleaned of spatially specific structured noise using the HCP’s multi-run (version 4.0) ICA-FIX for multi fMRI (multiple finger tasks) and linear trends without regressing out motion parameters. Somatotopic functional responses were estimated (first-level for participant P1 and second-level fixed-effect averaging of the two phase-encoding directions for participant P3) using a generalized linear model (GLM)-based fMRI analysis (Woolrich et al., 2001) on the grayordinate data space for each finger.

### Electrode Localization

The SEEG electrodes (PMT Corporation, Chanhassen, MN, United States) consisted of 16 contacts, cylinders with 2 mm length, 0.8 mm diameter, and 4.43 mm spacing (center to center). The HD-ECoG grids (PMT Corporation) consisted of 2 mm diameter flat contacts, in participant P1 with an 8 × 8 arrangement with 5 mm spacing (center to center) and in participant 2 with 16 × 16 contact arrangement with 4 mm spacing. Since both patients had clinical indications that required mapping of the sensorimotor cortex, task-based fMRI activation maps were used to guide electrode placement. For digital localization of the electrodes, we used the freely available iElvis toolbox, available at https://github.com/iELVis/ (Groppe et al., 2017). Briefly, the electrodes were manually localized using the software BioImage Suite^1^ on a postimplant CT which was co-registered using an affine transformation (6 degrees-of-freedom FLIRT; ^2^) to the pre-implantation 3T high-resolution T1w MRI. We used the FreeSurfer output from the HCP minimal processing pipeline (Glasser et al., 2013) to obtain the pial surface. The subdural HD-ECoG electrodes were projected to the smoothed pial surface. The smoothed pial surface, also called the outer smoothed surface, is generated by Freesurfer and wraps tightly around the gyral surfaces of the pial layer while bridging over the sulci. No correction was applied to SEEG electrode coordinates. To visualize the fMRI activation maps and the electrodes simultaneously, we used HCP Connectome Workbench^3^. Before importing the electrode coordinates into Connectome Workbench, we applied a RAS coordinate offset as follows –

*transformed_RAS_coordinates* = *Norig^∗^inv(Torig)^∗^RAS_coordinates*

where the transformation matrices *Norig* is obtained by *mri_info –vox2ras [subject]/mri/orig.mgz* and *Torig* is obtained by *mri_info –vox2ras-tkr [subject]/mri/orig.mgz*

The transformed coordinates were then imported as foci using the cortical surfaces into Connectome Workbench.

### Recording of Neural Activity

In addition to the clinical recording system, neural activity was recorded using a Neuroport System (Blackrock Microsystems, Salt Lake, Utah) with a sampling rate of 10 kHz while participants performed the various tasks. These tasks included hand/finger movements and mechanical stimulation of the fingertips of their hand using a von Frey filament (TouchTest Sensory Probes) of evaluator size, 3.61 (0.4 g). An electrode located in soft tissue lacking neural activity was used as the system ground. Subsequent analysis involved multiple steps to extract information regarding power modulation in different frequency bands. Signals from neighboring electrodes were subtracted in software to provide bipolar data with reduced noise. Non-overlapping Blackman windows of 200 ms in length were applied to the data, followed by a short Fast Fourier Transform (sFFT) for each window (with a resulting frequency resolution of 5 Hz) and a 1 s boxcar filter. The non-overlapping windowing approach was chosen to allow fast computation and to support artifact removal (in other ongoing studies where real-time processing is required and concurrent stimulation is present). The amplitude information at each frequency was then integrated across pre-selected frequency bands as follows: 0–10 (delta and theta), 10–15 (alpha/mu), 15–30 (beta), 30–100 (“gamma 1”), 100–500 (“gamma 2”), and 500–5,000 (“gamma 3”) Hz. These frequency ranges were selected to align with standard bands (as noted) and three gamma bands were assigned with increasing bandwidth to compensate for decreasing power density as frequency increases, thereby maintaining comparable signal amplitudes and quality for decoding. This produced integrated amplitude features (IAFs) for all bipolar recordings that were standardized by subtracting their mean and dividing by their standard deviation across the entire task.

### Task, Decoding, and Feature Selection Methods

Two different motor tasks and one sensory task were implemented in the study. The first motor task, called the “Open-Close Hand” task, included the participant opening their hand widely (with splayed fingers) and closing the hand (making a fist) when visually cued by an animated hand on a laptop (Figure 1). The second motor task, called the “Thumb-Middle Flexion” task involved flexing each digit (separately) in a sustained manner, mimicking the animated hand. The sensory task involved tapping the pads of three different digits (separately) using a von Frey filament (TouchTest Sensory Probes) of evaluator size, 3.61 (0.4 g), during each visual cue (not seen by the participant). In post-session analysis, epochs were created that aligned with each visual cue, starting at the cue onset and extending to 400 ms after the cue offset. All cue-aligned trials for each IAF were averaged to form a composite temporal response. To quantify the repeatability of potential features, an algorithm based on temporal correlation was used to compute the mean correlation coefficient (MCC) by averaging the correlation coefficients obtained for temporal responses for each trial with respect to the composite temporal response. Features were then selected based on their MCC value. The range of the MCC values chosen were 0.4 to 0.6, known as moderate correlation levels, as they led to improved decoding performance. MCC values outside this range tended to worsen the decoding performance. Selected features were then used to train an LSTM type RNN using Matlab and the Deep Learning Toolbox (R2019b, Update 4). Training parameters are as noted in Results section.

## Results

**FIGURE 2:**
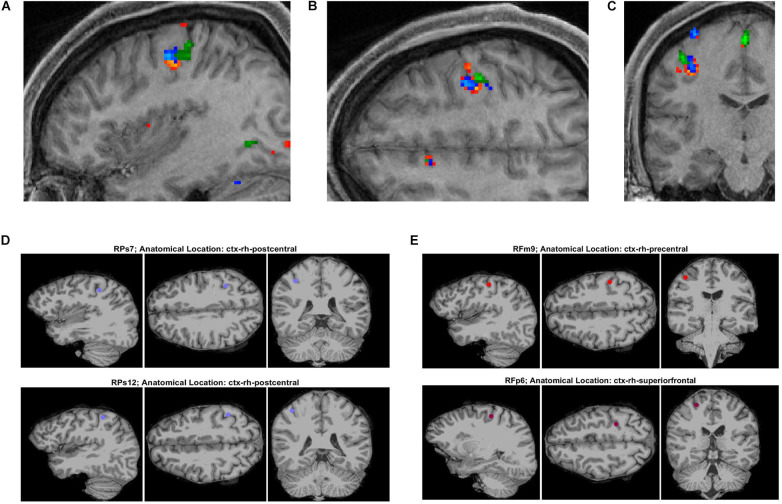
Functional MRI and post-surgery CT images for participant P3 related to finger tasks. **(A–C)** Functional MRI images in orthogonal planes (sagittal, transverse, and coronal) obtained during button press task for digits 1 (orange-red), 2 (blue), and 3 (green). **(D,E)** Post-surgical CT images of SEEG electrode sites found to have a repeatability metric (MCC) value greater than 0.6 (for time range of 0–4.4s with respect to cue onset) for flexion of digits 1 and 3.

Functional MRI images were obtained in participants P1 and P3 and post-surgical CT images documenting electrode locations were performed in all three participants. The fMRI images for the button press paradigm are shown in Figures 2A–C. The fMRI the peak activity was primarily located in the central sulcus for all three digit movements the participant performed during the task. Also shown in Figures 2D,E are post-surgical CT images highlighting electrodes that have signal features that were selected for decoding. The feature selection algorithm required that the MCC values for features to be used in decoding algorithm be greater than 0.6 (moderate correlation) during finger flexion tasks.

**FIGURE 3:**
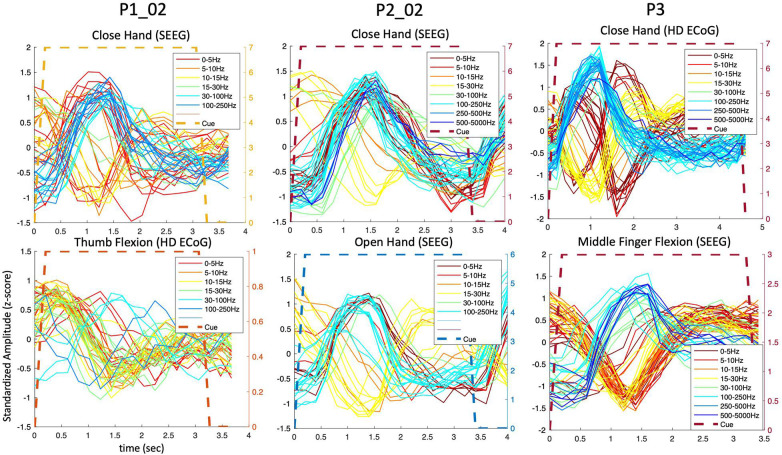
Predominantly phasic neural activity for sustained hand and finger movements in SEEG and ECoG recordings. Representative set of composite temporal responses across three study participants and different hand and finger movements are shown. In both the SEEG (depth) and HD ECoG (surface) recordings, the responses across a wide range of frequency bands are predominantly phasic in nature. The MCC (repeatability metric) was greater than 0.6 for all plots in this figure except in P1_02 where it was 0.5 for “Close hand (SEEG)” and 0.4 for “Thumb Flexion (HD ECoG).”

During the hand and finger movement tasks, phasic (transient) and phasic-tonic (transient-sustained) evoked responses were identified, using temporal correlation analysis, in all frequency bands analyzed across the 0 to 5,000 Hz range (as shown in Figure 3). Most evoked responses in the delta, theta, alpha/mu, and beta bands exhibited a decrease in amplitude during the pre-movement phase (after visual cue presentation to the participant), whereas an increase in power was observed in the gamma bands. An exception was observed in one participant (P3) where both increases and decreases were observed in the lower frequency bands. Also, it is readily seen in these results that the neural responses are largely phasic in nature (despite the sustained hand and finger movements performed) motivating the use of a LSTM network.

**FIGURE 4:**
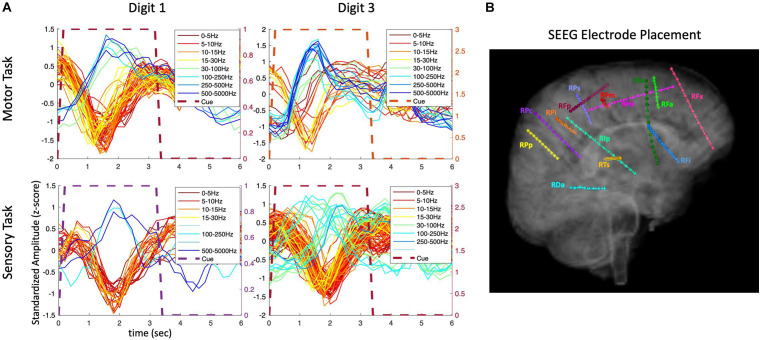
Amplitude modulation and electrode placement in P3_01 (SEEG electrodes). **(A)** Composite temporal patterns of amplitude modulation in various frequency bands for motor and sensory tasks. **(B)** Electrode placement in various regions of the brain.

To further examine the transient nature of the evoked responses, an analysis extended to the time period after the visual cue offset was performed. As shown in Figure 4, the composite temporal responses recorded from SEEG electrodes were observed not only after the cue onset, but also after the cue offset, in both motor and sensory tasks in participant P3. In the sensory tasks, mechanical tactile stimuli (rapid tapping) were presented throughout the entire cue period at the fingertips (center of pads).

**FIGURE 5:**
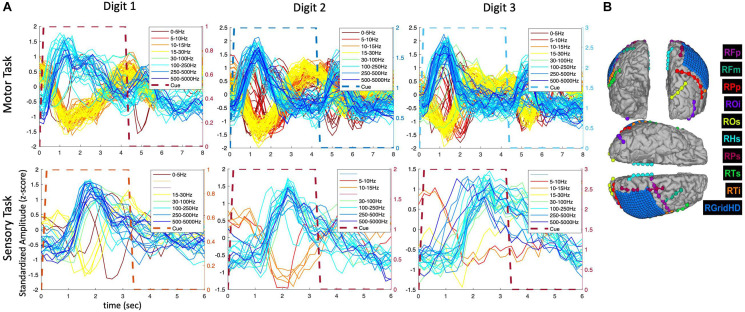
Amplitude modulation and electrode placement in P3 (ECoG and strip electrodes). **(A)** Averaged (across trials) temporal patterns of amplitude modulation in various frequency bands for motor and sensory tasks. **(B)** Electrode placement in various surface regions of the brain.

The extended time analysis was also performed for the HD ECoG recordings in the same participant (P3) during both motor and sensory tasks as shown in Figure 5. Similar phasic and phasic-tonic responses were observed but more features with high temporal correlation were identified (as the HD ECoG array was placed directly over the sensorimotor cortex). Interestingly, responses with more tonicity were observed on some digits, but which digit differed depending on whether a motor or sensory task was being performed.

Given that the evoked responses observed were primarily phasic in nature and had a wide variety of temporal shapes, and that stable features (features with high repeatability) are desirable for accurate decoding, temporal correlation analysis was introduced to quantify repeatability (stability) of potential features. It was hypothesized using temporal correlation-based feature selection would identify stable features (of any shape) and therefore improve decoding performance.

**FIGURE 6:**
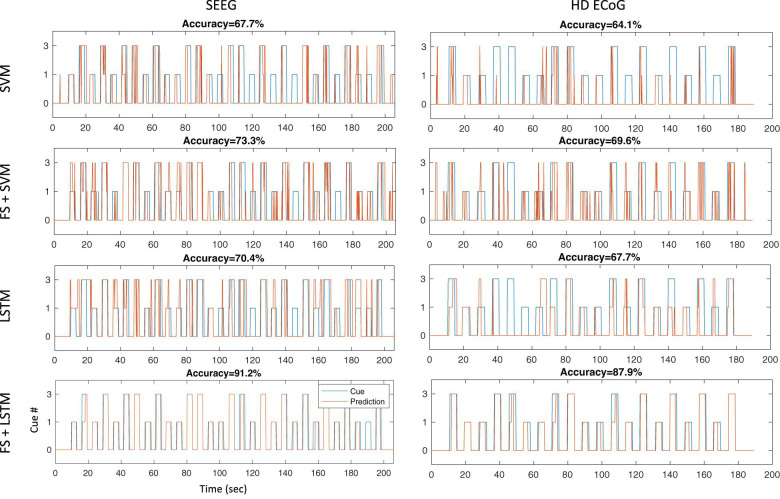
Decoding accuracies for different architectures during thumb and middle finger flexion task. Independent test sets results are shown for four different decoding architectures tested offline with SEEG and ECoG recordings in participant P3: the first row results are for a multi-class non-linear SVM using a gaussian kernel without feature selection, the second row are results for an architecture that performs repeatability-based feature selection (MCC > 0.6) and SVM, the third row shows results for a LSTM recurrent neural network without feature selection, and the last row shows results for an architecture using feature selection as described and a LSTM recurrent neural network.

As shown in Figure 6, using temporal correlation-based feature selection significantly improves decoding accuracy for both SVM and LSTM type algorithms when using SEEG or HD ECoG type electrode recordings in participant P3. This trend was also observed in the other participants. Note the task used for the HD ECoG recording involved three finger movements, but was reprocessed to remove the additional finger (index) cue to match the SEEG task for a more direct decoding performance comparison. In both SEEG and HD ECoG recordings, and for SVM or LSTM methods, decoding accuracy was improved when temporal correlation-based feature selection is used.

Another striking result shown in Figure 6 is the sustained output (“prediction”) from the LSTM network as compared to the fragmented SVM output/prediction during the sustained hand movements. This result offers support for our previous hypothesis that a LSTM approach may lead to sustained outputs and improved decoding accuracies during sustained movement tasks.

**FIGURE 7:**
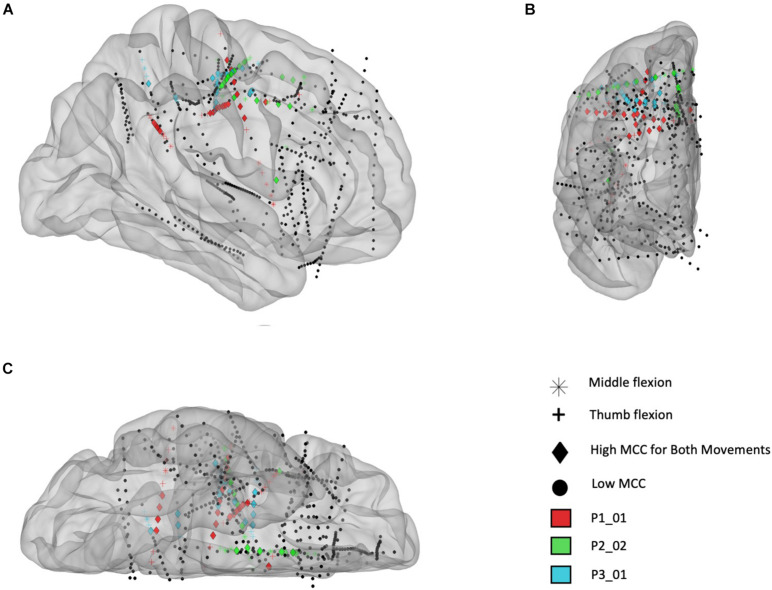
Electrode locations (right hemisphere) mapped to standard brain based on the Yeo 7 atlas for participants P1_01 (red), P2_02 (green), and P3_01 (blue). **(A)** Sagittal view from lateral perspective. **(B)** Coronal view from anterior perspective. **(C)** Transverse view from inferior perspective. The colored electrodes had high temporal correlation (MCC > 0.6) during a cued finger task including sustained thumb and middle flexion movements, separately, and the symbols mark which movement(s) had high MCC values: thumb flexion (cross), middle finger flexion (asterisk), or both (diamond). Note: electrodes are sometimes not inserted fully by the surgeon, therefore some contacts are located outside the brain (as seen in this figure).

In Figure 7 the electrode locations for all three participants (red = P1, green = P2, and blue = P3), mapped to a standard glass brain based on the Yeo 7 atlas (Yeo et al., 2011), are shown for the finger flexion task involving thumb flexion (+) and middle finger flexion (^∗^) prompted by visual cues. Temporal correlation analysis was performed and electrodes that yielded a MCC value greater than 0.6 (in one or more frequency bands) are shown using a colored symbol and a diamond shape is used to denote that the MCC value was greater than 0.6 for a given electrode during *both* the thumb and middle flexion cued movement periods. Overall, most of the electrodes with significant MCC values are in the sensorimotor areas for all three participants.

**FIGURE 8:**
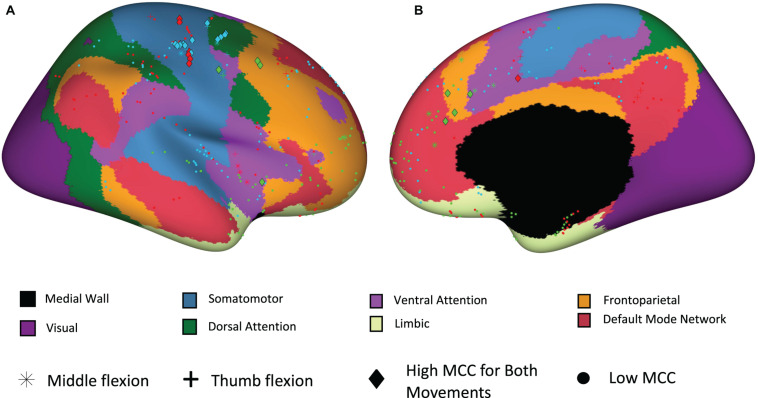
Electrode site locations with respect to functional networks for participants P1_01 (red), P2_02 (green), and P3_01 (blue). **(A)** Sagittal view from lateral perspective. **(B)** Sagittal view from medial perspective.

To further examine the location of electrodes with high temporal correlation, a functional network map was created. Electrodes within 3 mm of the cortical surface were snapped to the nearest point on the surface of the brain and shown on an inflated standard brain based on the Yeo 7 network atlas. As shown in Figure 8, most of the electrodes with high temporal correlation (MCC > 0.6) are located in the somatomotor, dorsal/ventral attention, and frontoparietal regions. Only two electrodes in the second subject (P2/green) have a high MCC value, but these are the only two electrodes implanted in this region (as confirmed by the lack of spherical green symbols).

Decoding results are shown in Table 1 for motor tasks where the participant performed sustained hand and finger movements including open (wide open of hand with splayed fingers), close hand (make a fist), and finger flexion (sustained flexion of individual fingers). The decoding accuracies achieved in SEEG recordings for open-close hand movements were comparable (with notably fewer recording sites for SEEG). For open-close hand movements, the sampling frequency for recordings in participant P1 was 1,024 Hz rather than the default 10 kHz rate due to system limitations at the time of the recordings. This reduced sampling rate was used for both SEEG and HD-ECoG recordings, therefore allowing direct comparison. In the thumb-middle flexion tasks, the decoding accuracy for SEEG recordings in participant 1 was significantly higher than that of the HD ECoG based recordings, even when the SEEG sampling rate was reduced to match the HD ECoG recording. In participant P3 for the thumb-middle flexion task the data (as noted previously) was reprocessed to remove the additional finger (index) cue to match the SEEG task for a more direct decoding performance comparison. The decoding accuracy in this task was found to be higher for the SEEG as compared to the HD-ECoG recording. Another interesting finding was that many SEEG electrode sites with high temporal correlation (MCC > 0.6), and therefore selected for decoding, were found to be in white matter. In fact, for the open-close hand task, over fifty percent of these sites were in white matter for two of the three participants (16 of 27 sites for P2_02, 9 of 16 sites for P3_01, and 3 of 19 sites for P1_03).

**TABLE 1:** Decoding performance (mean ± SD) for sustained hand movements when using a recurrent neural network (LSTM type) with repeatability-based feature selection.

Participant	Open-close hand		Thumb-middle flexion	
	SEEG	HD ECoG	SEEG	HD ECoG
P1	77.48 ± 3.65%^1^ (19 sites, 3 in sensorimotor)	79.98 ± 0.48%^1^ (30 sites)	83.92 ± 2.25%^2^ (39 sites)	70.06 ± 1.35%^2^ (22 sites)
P2_02	91.61% ± 1.29% (27 sites)	N/A	84.29% ± 1.46% (33 sites)	N/A
P3	92.04 ± 1.51% (16 sites)	88.41 ± 3.36% (43 sites)	91.69 ± 0.49% (37 sites)	87.40 ± 1.03% (60 sites)

*^1^The sampling rate used was 1,024 Hz (due to system limitations at time of recording). ^2^The sampling rate used was 512 Hz (due to system limitations at time of recording). MCC threshold ranged from 0.55 to 0.6, the number of hidden units ranged from 400 to 600, and the patience ranged from 2 to 7.*

During the tactile stimuli task where a mechanical filament was used to repeatedly tap the pads of digits 1, 2, and 3, decoding results were obtained for SEEG and/or HD ECoG recordings. The decoding accuracies when using an LSTM based algorithm with temporal correlation-based feature selection for these different recording modalities and the three different participants are shown in Table 2. To allow decoding performance comparisons with an equal number of cues, the HD ECoG data sets for P1_03 and P3 were reprocessed to remove additional tapping locations. The decoding accuracy results for SEEG recordings were significantly lower in participant P1_03 than those obtained with HD ECoG recordings, however, 28 of the 31 recording sites were located outside the sensory area of the brain. In participant P3, the decoding accuracy for SEEG-based recordings were slightly lower as well, but with fewer recording sites (21 versus 30 required for decoding of the HD ECoG recording).

**TABLE 2:** Decoding performance (mean ± SD) for tactile stimuli (tapping) applied to thumb, index, and middle finger pads when using a LSTM type recurrent neural network with a feature selection algorithm based on temporal correlation.

Participant	SEEG	HD ECoG
P1_03	62.21 ± 1.50%^1^ (31 sites)	70.04 ± 2.86%^1^ (45 sites)
P2_02	80.64 ± 1.64% (9 sites)	N/A
P3	78.64 ± 0.64% (21 sites)	83.49 ± 0.72% (30 sites)

*^1^Sampling rate used was 1,024 Hz (due to system limitations at time of recording).*

## Discussion

In this study we showed minimally invasive SEEG electrodes can record stable and information-rich neural signals related to movement and tactile sensation. Using a temporal correlation-based feature selection method, we identified and extracted repeating neural patterns that consistently occurred during motor and sensory events. These features were used as inputs to a LSTM type recurrent neural network which was able to reliably and accurately predict finger movement and focal tactile stimuli presented at the pads of the fingers.

High decoding accuracy was demonstrated while using SEEG methods in multiple participants across multiple tasks and shown to be comparable to the decoding performances achieved with HD ECoG. This was unexpected given the relatively low spatial resolution of SEEG electrodes (4.43 mm site spacing) and that there were fewer electrodes placed in the sensorimotor area as compared to the HD-ECoG grid electrodes. Furthermore, very few studies have demonstrated the application of SEEG in neural decoding and in the BCI field. In fact, the authors are not aware of any other previous study demonstrating the use of SEEG/depth electrodes for neural decoding of both motor and sensory stimuli events.

During this study it was readily observed that the neural responses were largely phasic in nature, despite the sustained hand and finger movements performed. This motivated our use of a LSTM network since it can produce sustained outputs while receiving transient inputs (due to the memory cells inherent in their network structure). The delta, theta, alpha/mu, and beta bands exhibited a decrease in amplitude, along with an increase in the gamma band, during the pre-movement phase. These results were expected, however, some increases in feature amplitudes in the lower frequency bands for participant (P3) occurred which may be due to some of the raw evoked potential component (0 Hz) being incorporated in the low frequency band processing.

Another interesting finding in this study is that many electrode sites producing useful features for decoding were in white matter. This was surprising since white matter does not contain neurons and is usually considered electrically neutral. Other studies, however, have found that recorded white matter signals contain a mixture of activity which appears to be from both nearby and distant gray matter activity (Mercier et al., 2017). High density EEG studies have also shown that sources involving sensorimotor processing can be located in white matter areas (Melnik et al., 2017). Furthermore, recent studies have also shown SEEG recording sites located in white matter can contribute to accurate decoding (Huang et al., 2019, 2021). We feel this area is ripe for further exploration and plan to perform more recordings that include electrode sites in the white matter in the future.

This study had limitations including the number of participants and small task differences. In the future, expanding the study to include additional participants will allow further mapping of sulcal and white matter areas which are less charted than the gyri. In the current study reported here, not all tasks were completed for all participants due to session time constraints, therefore some decoding performance comparisons are not available. Despite this, the data suggest SEEG recordings, combined with the methods presented, can achieve high decoding accuracies using relatively few electrode sites. Furthermore, patients were taken off of their anti-seizure medications before recording/decoding sessions were started, however, residual effects may have reduced neuronal activity and impacted reaction times. Lastly, the brain atlas we used for producing Figure 8 did not delineate between motor and sensory areas, so we therefore plan to utilize an alternate atlas in future studies.

With the clear advantage of being minimally invasive, SEEG electrodes may be a viable option for use in brain-computer interface systems and specifically in a bidirectional neural bypass for paralysis applications. Although SEEG electrodes have been limited to acute use, similarly constructed depth electrodes, such as those used in deep brain stimulation for Parkinson’s disease, which have been implanted for multiple years, and some investigational devices have recording electrodes such as the Summit^TM^ RC + S developed by Medtronic (Stanslaski et al., 2018).

The temporal correlation feature selection method was also found to identify locations within the brain involved in processing sensory stimuli that corresponded to the locations identified through cortical stimulation methods (Chandrasekaran et al., 2020). Passive methods such as this that do not involve electrical stimulation are attractive for mapping procedures in epileptic patients as they reduce the risk of inducing a seizure. The methods demonstrated in this study have many potential applications in mapping, movement and sensory restoration, and augmentative communication for patients living with paralysis, sensory loss, ALS, epilepsy, brain injury, and many other neurological conditions.

## Conclusion

Here we explored the viability of using minimally invasive SEEG methods and electrodes for decoding neural activity related to motor and sensory events. As observed in ECoG recordings, neural signals produced by SEEG electrodes are primarily phasic (transient) in nature, even during sustained motor tasks. When a feature selection method based on temporal correlation (a measure of feature repeatability) was implemented, decoding accuracy increased with both SVM and LSTM type recurrent neural network approaches. Furthermore, the overall decoding accuracy for SEEG recordings was comparable to the performance observed in ECoG recordings. Our findings support that SEEG can be an effective approach for neural decoding and for use in brain-computer interface systems. Finally, this minimally invasive approach reduces risk and may become the preferred approach for many BCI applications including restoration of movement and tactile sensation in impaired users.

## Data Availability Statement

The raw data supporting the conclusions of this article will be made available by the authors, without undue reservation.

## Ethics Statement

The studies involving human participants were reviewed and approved by Northwell Health Institutional Review Board. The patients/participants provided their written informed consent to participate in this study.

## Author Contributions

CB, NB, SC, and SB designed the study. JK and JX designed and performed the fMRI procedures and analyzed the data. AM performed the SEEG leads and HD-ECoG grid implantations. NM and EE digitized and co-registered the electrode locations. NB, SC, SB, JH, RR, and CB performed all the experiments. CB analyzed data from these experiments. MG and JX provided key insights into cortical anatomy, help using the workbench software and generating relevant figures for the manuscript, along NM. All authors contributed toward interpreting the results of the experiments. CB wrote the initial draft of the manuscript. All authors provided critical review, edits, and approval of the final manuscript.

## Conflict of Interest

CB has ownership interests in Neuvotion, LLC and is an inventor on multiple patents in the related field of neuroprosthetics. The remaining authors declare that the research was conducted in the absence of any commercial or financial relationships that could be construed as a potential conflict of interest.

## Publisher’s Note

All claims expressed in this article are solely those of the authors and do not necessarily represent those of their affiliated organizations, or those of the publisher, the editors and the reviewers. Any product that may be evaluated in this article, or claim that may be made by its manufacturer, is not guaranteed or endorsed by the publisher.

## Footnotes

1
http://www.bioimagesuite.org

2
www.fmrib.ox.ac.uk/fsl

3
https://www.humanconnectome.org/software/connectome-workbench

## References

[B1] Akbari H. Khalighinejad B. Herrero J. L. Mehta A. D. Mesgarani N. (2019). Towards reconstructing intelligible speech from the human auditory cortex. *Sci. Rep.* 9:874. 10.1038/s41598-018-37359-z PMC6351601 30696881

[B2] Armour B. S. Courtney-Long E. A. Fox M. H. Fredine H. Cahill A. (2016). Prevalence and causes of paralysis - United States, 2013. *Am. J. Public Health* 106 1855–1857. 10.2105/ajph.2016.303270 27552260 PMC5024361

[B3] Bengio Y. Simard P. Frasconi P. (1994). Learning long-term dependencies with gradient descent is difficult. *IEEE Trans. Neural Netw.* 5 157–166. 10.1109/72.279181 18267787

[B4] Bouton C. E. Shaikhouni A. Annetta N. V. Bockbrader M. A. Friedenberg D. A. Nielson D. M. (2016). Restoring cortical control of functional movement in a human with quadriplegia. *Nature* 533 247–250. 10.1038/nature17435 27074513

[B5] Cardinale F. Cossu M. Castana L. Casaceli G. Schiariti M. P. Miserocchi A. (2013). Stereoelectroencephalography: surgical methodology, safety, and stereotactic application accuracy in 500 procedures. *Neurosurgery* 72 353–366. 23168681 10.1227/NEU.0b013e31827d1161

[B6] Carmena J. M. Lebedev M. A. Crist R. E. O’Doherty J. E. Santucci D. M. Dimitrov D. F. (2003). Learning to control a brain-machine interface for reaching and grasping by primates. *PLoS Biol*. 1:E42. 10.1371/journal.pbio.0000042 14624244 PMC261882

[B7] Chandrasekaran S. Bickel S. Herrero J. L. Kim J. W. Markowitz N. Espinal E. (2020). Evoking highly focal percepts in the fingertips through targeted stimulation of sulcal regions of the brain for sensory restoration. *MedRxiv [preprint]* 10.1101/2020.11.06.20217372 PMC8884403 34358704

[B8] Chen C. Shin D. Watanabe H. Nakanishi Y. Kambara H. Yoshimura N. (2014). Decoding grasp force profile from electrocorticography signals in non-human primate sensorimotor cortex. *Neurosci. Res*. 83 1–7. 10.1016/j.neures.2014.03.010 24726922

[B9] Chowdhury R. H. Glaser J. I. Miller L. E. (2020). Area 2 of primary somatosensory cortex encodes kinematics of the whole arm. *ELife* 9:e48198. 10.7554/eLife.48198 PMC6977965 31971510

[B10] Flesher S. N. Collinger J. L. Foldes S. T. Weiss J. M. Downey J. E. Tyler-Kabara E. C. (2016). Intracortical microstimulation of human somatosensory cortex. *Sci. Transl. Med.* 8:361ra141. 10.1126/scitranslmed.aaf8083 27738096

[B11] Flesher S. N. Downey J. E. Weiss J. M. Hughes C. L. Herrera A. J. Tyler-Kabara E. C. (2021). A brain-computer interface that evokes tactile sensations improves robotic arm control. *Science* 372 831–836. 10.1126/science.abd0380 34016775 PMC8715714

[B12] Flint R. D. Rosenow J. M. Tate M. C. Slutzky M. W. (2017). Continuous decoding of human grasp kinematics using epidural and subdural signals. *J. Neural Eng.* 14:016005. 10.1088/1741-2560/14/1/016005 PMC5528155 27900947

[B13] Glasser M. F. Sotiropoulos S. N. Wilson J. A. Coalson T. S. Fischl B. Andersson J. L. (2013). The minimal preprocessing pipelines for the Human Connectome Project. *NeuroImage* 80 105–124. 10.1016/j.neuroimage.2013.04.127 23668970 PMC3720813

[B14] Groppe D. M. Bickel S. Dykstra A. R. Wang X. Mégevand P. Mercier M. R. (2017). iELVis: an open source MATLAB toolbox for localizing and visualizing human intracranial electrode data. *J. Neurosci. Methods* 281 40–48. 10.1016/j.jneumeth.2017.01.022 28192130

[B15] Hochberg L. R. Serruya M. D. Friehs G. M. Mukand J. A. Saleh M. Caplan A. H. (2006). Neuronal ensemble control of prosthetic devices by a human with tetraplegia. *Nature* 442 164–171. 10.1038/nature04970 16838014

[B16] Hosman T. Vilela M. Milstein D. Kelemen J. N. Brandman D. M. Hochberg L. R. (2019). “BCI decoder performance comparison of an LSTM recurrent neural network and a Kalman filter in retrospective simulation,” in *Proceeding of the International IEEE/EMBS Conference on Neural Engineering, NER*, (San Francisco, CA).

[B17] Huang W. Yu T. Xiao J. Guo Q. Li Y. (2019). “A P300-based brain computer interface using stereo-electroencephalography signals,” in *Proceeding of the 2019 41st Annual International Conference of the IEEE Engineering in Medicine and Biology Society (EMBC)*, (IEEE), 3062–3066. 10.1109/EMBC.2019.8857724 31946534

[B18] Huang W. Zhang P. Yu T. Gu Z. Guo Q. Li Y. (2021). A P300-Based BCI system using stereoelectroencephalography and its application in a brain mechanistic study. *IEEE Trans. Bio-Med. Eng.* 68 2509–2519. 10.1109/TBME.2020.3047812 33373294

[B19] Jiang T. Pellizzer G. Asman P. Bastos D. Bhavsar S. Tummala S. (2020). Power modulations of ECoG alpha/beta and gamma bands correlate with time-derivative of force during hand grasp. *Front. Neurosci*. 14:100. 10.3389/fnins.2020.00100 32116533 PMC7033626

[B20] Krusienski D. J. Shih J. J. (2011). Control of a brain-computer interface using stereotactic depth electrodes in and adjacent to the hippocampus. *J. Neural Eng*. 8:025006. 10.1088/1741-2560/8/2/025006 PMC3150521 21436521

[B21] Kubánek J. Miller K. J. Ojemann J. G. Wolpaw J. R. Schalk G. (2009). Decoding flexion of individual fingers using electrocorticographic signals in humans. *J. Neural Eng.* 6:066001. 10.1088/1741-2560/6/6/066001 PMC3664231 19794237

[B22] Li D. Han H. Xu X. Ling Z. Hong B. (2017). “Minimally invasive brain computer interface for fast typing,” in *Proceeding of the International IEEE/EMBS Conference on Neural Engineering, NER*, (IEEE).

[B23] Li G. Jiang S. Xu Y. Wu Z. Chen L. Zhang D. (2017). “A preliminary study towards prosthetic hand control using human stereo-electroencephalography (SEEG) signals,” in *Proceeding of the International IEEE/EMBS Conference on Neural Engineering, NER*, (IEEE).

[B24] Melnik A. Hairston W. D. Ferris D. P. König P. (2017). EEG correlates of sensorimotor processing: independent components involved in sensory and motor processing. *Sci. Rep.* 7:4461. 10.1038/s41598-017-04757-8 PMC5493645 28667328

[B25] Mercier M. R. Bickel S. Megevand P. Groppe D. M. Schroeder C. E. Mehta A. D. (2017). Evaluation of cortical local field potential diffusion in stereotactic electro-encephalography recordings: a glimpse on white matter signal. *NeuroImage* 147 219–232. 10.1016/j.neuroimage.2016.08.037 27554533

[B26] Murphy B. A. Miller J. P. Gunalan K. Ajiboye A. B. (2016). Contributions of subsurface cortical modulations to discrimination of executed and imagined grasp forces through stereoelectroencephalography. *PLoS One* 11:e0150359. 10.1371/journal.pone.0150359 26963246 PMC4786254

[B27] Rule M. E. Loback A. R. Raman D. V. Driscoll L. N. Harvey C. D. O’leary T. (2020). Stable task information from an unstable neural population. *ELife* 9:e51121. 10.7554/eLife.51121 PMC7392606 32660692

[B28] Setsompop K. Gagoski B. A. Polimeni J. R. Witzel T. Wedeen V. J. Wald L. L. (2012). Blipped-controlled aliasing in parallel imaging for simultaneous multislice echo planar imaging with reduced g-factor penalty. *Magnetic Resonance Med*. 67 1210–1224. 10.1002/mrm.23097 21858868 PMC3323676

[B29] Shewalkar A. Nyavanandi D. Ludwig S. A. (2019). Performance evaluation of deep neural networks applied to speech recognition: RNN, LSTM and GRU. *J. Artificial Intelligence Soft Computing Res.* 9 235–245. 10.2478/jaiscr-2019-0006

[B30] Shih J. J. Krusienski D. J. (2012). Signals from intraventricular depth electrodes can control a brain-computer interface. *J. Neurosci. Methods* 203 311–314. 10.1016/j.jneumeth.2011.10.012 22044847 PMC3246120

[B31] Shin D. Watanabe H. Kambara H. Nambu A. Isa T. Nishimura Y. (2012). Prediction of muscle activities from electrocorticograms in primary motor cortex of primates. *PLoS One*. 7:e47992. 10.1371/journal.pone.0047992 23110153 PMC3480494

[B32] Stanslaski S. Herron J. Chouinard T. Bourget D. Isaacson B. Kremen V. (2018). A chronically implantable neural coprocessor for investigating the treatment of neurological disorders. *IEEE Trans. Biomed. Circuits Syst*. 12 1230–1245. 10.1109/tbcas.2018.2880148 30418885 PMC6415546

[B33] Stricsek G. Lang M. J. Wu C. (2018). “Stereoelectroencephalography (sEEG) versus grids and strips,” in *Functional Neurosurgery and Neuromodulation* (Amsterdam: Elsevier), 113–120.

[B34] Vadera S. Marathe A. R. Gonzalez-Martinez J. Taylor D. M. (2013). Stereoelectroencephalography for continuous two-dimensional cursor control in a brain-machine interface. *Neurosurg. Focus* 34:E3. 10.3171/2013.3.FOCUS1373 23724837

[B35] Woolrich M. W. Ripley B. D. Brady M. Smith S. M. (2001). Temporal autocorrelation in univariate linear modeling of FMRI data. *NeuroImage* 14 1370–1386. 10.1006/nimg.2001.0931 11707093

[B36] Xu J. Moeller S. Auerbach E. J. Strupp J. Smith S. M. Feinberg D. A. (2013). Evaluation of slice accelerations using multiband echo planar imaging at 3T. *NeuroImage* 83 991–1001. 10.1016/j.neuroimage.2013.07.055 23899722 PMC3815955

[B37] Yeo B. T. T. Krienen F. M. Sepulcre J. Sabuncu M. R. Lashkari D. Hollinshead M. (2011). The organization of the human cerebral cortex estimated by intrinsic functional connectivity. *J. Neurophysiol.* 106 1125–1165. 10.1152/jn.00338.2011 21653723 PMC3174820

[B38] Zhang M. Schwemmer M. A. Ting J. E. Majstorovic C. E. Friedenberg D. A. Bockbrader M. A. (2018). Extracting wavelet based neural features from human intracortical recordings for neuroprosthetics applications. *Bioelectronic Med.* 4:11. 10.1186/s42234-018-0011-x PMC7098253 32232087

